# Insights Into the Biodegradation of Lindane (γ-Hexachlorocyclohexane) Using a Microbial System

**DOI:** 10.3389/fmicb.2020.00522

**Published:** 2020-03-27

**Authors:** Wenping Zhang, Ziqiu Lin, Shimei Pang, Pankaj Bhatt, Shaohua Chen

**Affiliations:** ^1^State Key Laboratory for Conservation and Utilization of Subtropical Agro-bioresources, Guangdong Province Key Laboratory of Microbial Signals and Disease Control, Integrative Microbiology Research Centre, South China Agricultural University, Guangzhou, China; ^2^Guangdong Laboratory for Lingnan Modern Agriculture, Guangzhou, China

**Keywords:** lindane, biodegradation, metabolic pathway, mechanisms, bioremediation

## Abstract

Lindane (γ-hexachlorocyclohexane) is an organochlorine pesticide that has been widely used in agriculture over the last seven decades. The increasing residues of lindane in soil and water environments are toxic to humans and other organisms. Large-scale applications and residual toxicity in the environment require urgent lindane removal. Microbes, particularly Gram-negative bacteria, can transform lindane into non-toxic and environmentally safe metabolites. Aerobic and anaerobic microorganisms follow different metabolic pathways to degrade lindane. A variety of enzymes participate in lindane degradation pathways, including dehydrochlorinase (LinA), dehalogenase (LinB), dehydrogenase (LinC), and reductive dechlorinase (LinD). However, a limited number of reviews have been published regarding the biodegradation and bioremediation of lindane. This review summarizes the current knowledge regarding lindane-degrading microbes along with biodegradation mechanisms, metabolic pathways, and the microbial remediation of lindane-contaminated environments. The prospects of novel bioremediation technologies to provide insight between laboratory cultures and large-scale applications are also discussed. This review provides a theoretical foundation and practical basis to use lindane-degrading microorganisms for bioremediation.

## Introduction

**FIGURE 1:**
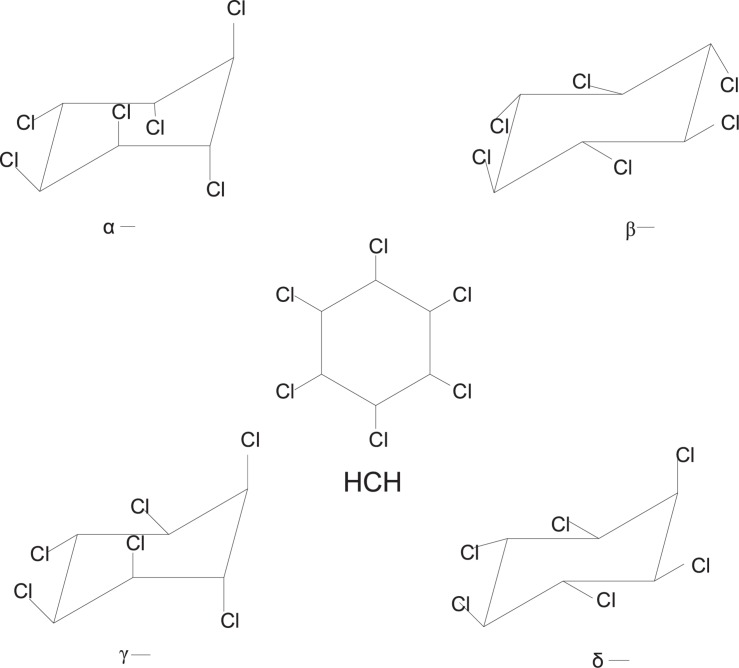
Chemical structures of four hexachlorocyclohexane (HCH) isomers.

Lindane (γ-hexachlorocyclohexane) is a broad-spectrum organochlorine pesticide belonging to the chlorinated hydrocarbon family. It was synthesized after the Second World War until the 1990s (Saez et al., 2015; Dominguez et al., 2018b). Four major isomers (Figure 1) are formed (α, β, γ, and δ) during the production of hexachlorocyclohexane (HCH), however, only the γ isomers possess insecticidal properties (Vijgen et al., 2019). For the last seven decades, lindane insecticide has been extensively used worldwide on fruits, vegetables, forest crops, animals, and on animal premises (Jung et al., 2018; Singh and Singh, 2019a). The insecticidal activity of lindane is due to its excitatory action on the nervous system (Sharma et al., 2010; Nolan et al., 2012). Lindane primarily acts on the γ-aminobutyric acid (GABA) receptor/chloride ionophore complex, which leads to hyperexcitation of the central nervous system (CNS) and results in paralysis, convulsions, and even death (Mladenović et al., 2010; Agrahari et al., 2019). Fractional crystallization is used for the extraction and purification of lindane from the production mixture (Padhi and Pati, 2016). Lindane production is inefficient, as the production of one ton of lindane creates approximately 8–12 tons of other waste isomers, mainly composed of α- and β-hexachlorocyclohexane (Vijgen et al., 2011).

Lindane can persist in the environment due to its high lipid solubility and chemical stability and can migrate over long distances to cause widespread contamination (Benimeli et al., 2008). The reported half-lives for lindane in soil and water were reported to be 708 and 2292 days, respectively (Beyer and Matthies, 2001). Generally, lindane soil residues enter the food chain and concentrate in the fat tissues of humans and animals causing major health hazards (Nolan et al., 2012). The lipid solubility of lindane increases its toxicity to humans and animals. Lindane has been classified as a carcinogen and endocrine disrupter, and it possesses mutagenic, genotoxic, and teratogenic effects (Markman et al., 2007; Luo et al., 2016; Muñiz et al., 2017).

In 2009, due to the environmental persistency and bioaccumulation potential, lindane, along with its α and β isomers, was included in the Stockholm Convention list of persistent organic pollutants (POPs) (Wacławek et al., 2016), and it has been banned or severely restricted. To implement the Stockholm Convention on POPs, the Ministry of Ecology and Environment of China has prohibited the production, circulation, application, import, and export of lindane since 26 March 2019 (Wang, 2019). Lindane is still being used in some developing countries for agricultural and public health purposes due to its low cost and versatility in pest control (Abhilash and Singh, 2010). Currently, India is the largest producer and consumer of lindane in the world (Sagar and Singh, 2011). Continuous applications and indiscriminate industrial production have led to widespread lindane contamination of soils in the country (Vankar and Ramashanker, 2011). Over the years, only a small portion of the related legal problems were addressed in the European Union (EU) or globally. Large stockpiles of lindane and its wastes are still present in the soil, which represents a large contaminant reservoir (Vijgen et al., 2019). Therefore, lindane degradation technologies must be developed to decontaminate the polluted soil and water.

**FIGURE 2:**
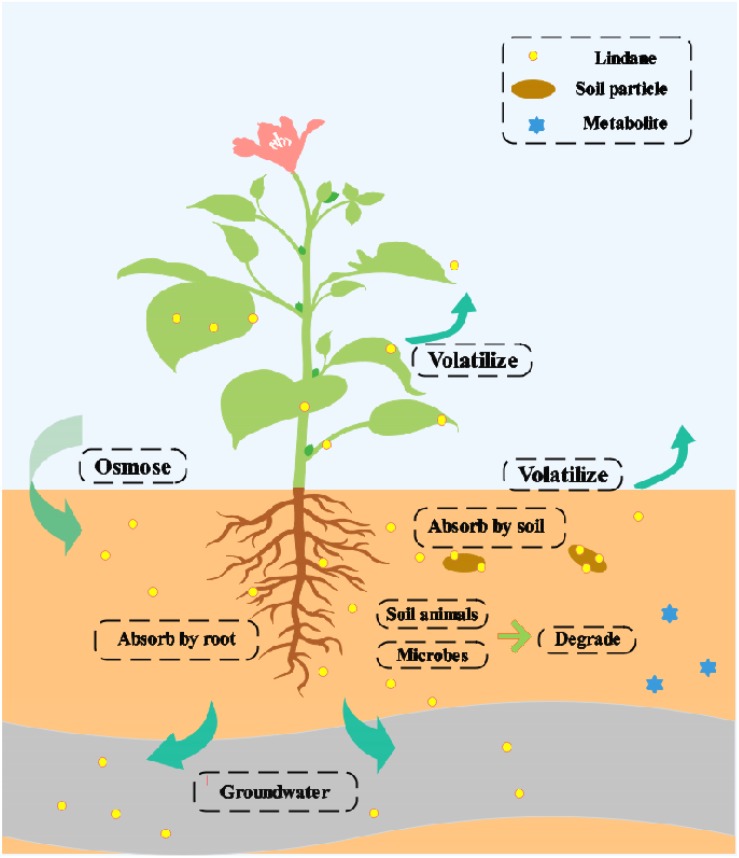
Migration and transformation of lindane in soil.

The migration and transformation of lindane in the soil is presented in Figure 2. Several factors control the lindane distribution and fate in the fields, including (a) loss by volatilization into the atmosphere, (b) loss through surface runoff, and (c) degradation by soil microbes and animals (Lal et al., 2010; Alvarez et al., 2012; Giri et al., 2014). The natural degradation of lindane is a very slow process and varies according to the environmental conditions (Elcey and Kunhi, 2009). Currently, a variety of treatment methods are available for the removal of lindane, but these all have certain drawbacks (Nagpal and Paknikar, 2006; Nagpal et al., 2008).

Physicochemical methods, such as incineration, electro-oxidation, and microwave-induced or chemical oxidation, are not eco-friendly and require large and expensive infrastructure (Liu et al., 2015; Zhan et al., 2020). They also generate highly toxic compounds during the degradation process (Manickam et al., 2006; Usman et al., 2014; Dominguez et al., 2018a, b; Jung et al., 2018; Huang et al., 2019). However, the microbial degradation of lindane contamination is attracting increasing attention as a less expensive and more environmentally friendly alternative to conventional treatment methods (Chen et al., 2015; Arora et al., 2017; Bajaj et al., 2017; Cycoń et al., 2017; Zhang H. et al., 2018; Bhatt et al., 2019a). The first report regarding the microbial degradation of lindane was published in 1967. Subsequently, numerous studies reported potential microorganisms for the efficient and rapid bioremediation of lindane-polluted environments (Sangwan et al., 2012; Githinji, 2015; Kumar et al., 2016). However, the literature lacks reviews regarding microbial resources and the mechanism of lindane degradation.

In this review, we emphasize the role of microbial degradation for large-scale bioremediation of lindane contamination. We compile detailed information about the microbial resources, mechanisms, and important enzymes involved in lindane degradation, and the aerobic and anaerobic metabolic pathways are also compared. Additionally, a brief account of recent lindane bioremediation methods is given. This review provides novel insights into the future of the large-scale bioremediation of lindane.

## Toxicity of Lindane

The extensive applications of lindane over the decades resulted in a wide distribution that affects the biota, and lindane is now considered one of the most hazardous POPs (Tsygankov et al., 2019). The chemical persistence and lipophilicity of lindane enable it to accumulate and biomagnify in food chains. On 27 October 2017, the World Health Organization published a list of carcinogens where lindane was categorized as a Group 1 carcinogen (Singh and Singh, 2019a).

Lindane is the most acutely toxic HCH isomer that affects the central nervous system and endocrine system. Lindane is attributed to various adverse health effects in humans, including immunosuppressive, oxidative, proinflammatory, carcinogenic, neurotoxic, and hormone-disruption effects, and even convulsions and death (Vega et al., 2016). The first evidence of death by lindane toxicity was reported in 1953 (Nolan et al., 2012). Due to the lipophilicity, lindane can infiltrate and accumulate in the brain, human breast milk, and other organs that are rich in fat. Epidemiological and agricultural health studies reported that lindane exposure could be associated with the cancers of the breast, prostate, lung, stomach, colon, rectum, and bladder (Jayaraj et al., 2016; Abolhassani et al., 2019; Mortazavi et al., 2019).

Broad applications of lindane pose serious threats to the environment. Once released, lindane can disseminate to all environmental compartments. Compared with other organochlorine pesticides, lindane is ubiquitous due to the high water solubility and volatility (Zhang et al., 2010). Lindane has a strong adsorption tendency in organic materials; therefore, it is long-lived and can be transported for long-ranges in the environment (Vega et al., 2016). Lindane contaminants have leached underground for decades, polluting both the surface water and groundwater (Weber et al., 2013). Lindane is a threat to a wide range of environments as it bioaccumulates in microorganisms, invertebrates, fish, birds, and mammals (Vijgen et al., 2018) and threatens human health through the food chain. The microbial community structure has a unique response to pesticide pollution, and recent research found that microbial diversity, richness, and structure undergo changes with lindane concentration (Sun et al., 2019).

## Potential Microorganisms in Lindane Degradation

The bacterial degradation of lindane and other xenobiotics are widely reported (Chen et al., 2012, 2013; Yang et al., 2018; Zhang H. et al., 2018; Bhatt et al., 2020c). Bacterial cells use organic pollutants as a sole source of carbon and nitrogen (Chen et al., 2011, 2014; Zhan et al., 2018b; Lin et al., 2020). Currently, a number of lindane-degrading bacterial strains have been screened, enriched, and domesticated (Table 1), including *Microbacterium* (Singh and Singh, 2019b), *Paracoccus* (Sahoo et al., 2019), *Achromobacter* (Singh and Singh, 2019a), *Burkholderia* (Kumar, 2018), *Rhodococcus* (Egorova et al., 2017), *Chromohalobacter* (Bajaj et al., 2017), *Kocuria* (Kumar et al., 2016), *Staphylococcus* (Kumar et al., 2016), *Streptomyces* (Sineli et al., 2016), *Arthrobacter* (De Paolis et al., 2013), *Azotobacter* (Anupama and Paul, 2009), *Sphingomonas* (Manickam et al., 2008), *Xanthomonas* (Manickam et al., 2010), *Pseudomonas* (Kumar et al., 2011), *Pseudoarthrobacter* (Nagpal and Paknikar, 2006), *Klebsiella* (Nagpal and Paknikar, 2006), *Pleurotus* (Dritsa and Rigas, 2013), *Fusarium* (Sagar and Singh, 2011), and *Actinobacteria* (Cuozzo et al., 2017).

**TABLE 1:** Lindane degradation by various microorganisms.

Species	Microorganism	Concentration of lindane	Degradation rate	Sources	References
Bacteria	*Microbacterium* sp. P27	50 mg/L	82.7% in 15 days	*Phragmites karka*	Singh and Singh, 2019b
	*Paracoccus* sp. NITDBR1	100 mg/L	90% in 8 days	Agricultural field	Sahoo et al., 2019
	*Achromobacter* sp. strain A3	50 mg/L	88.7% in 15 days	*Acorus calamus*	Singh and Singh, 2019a
	*Burkholderia* sp. IPL04	100 mg/L	98% in 8 days	Soil	Kumar, 2018
	*Rhodococcus wratislaviensis* Ch628	200 mg/L	32.3% in 5 days	Soil	Egorova et al., 2017
	*Chromohalobacter* sp. LD2	50 mg/L	89.6% in 7 days	Soil	Bajaj et al., 2017
	*Kocuria* sp. DAB-1Y	10 mg/L	94% in 8 days	Soil	Kumar et al., 2016
	*Staphylococcus* sp. DAB-1W	10 mg/L	98% in 8 days	Soil	Kumar et al., 2016
	*Streptomyces* sp. M7	1.66 mg/L	45% in 7 days	Sediment	Sineli et al., 2016
	*Arthrobacte fluorescens*	100 mg/L	40% in 72 h	Soil	De Paolis et al., 2013
	*Arthrobacter giacomelloi*	100 mg/L	56% in 72 h	Soil	De Paolis et al., 2013
	*Azotobacter chroococcum*	10 mg/L	Almost complete in 6 days	Farm fields	Anupama and Paul, 2009
	*Sphingomonas* sp. NM05	100 mg/L	90% in 7 days	Soil	Manickam et al., 2008
	*Xanthomonas* sp. ICH12	100 mg/L	100% in 8 days	Soil	Manickam et al., 2010
	*Pseudomonas aeruginosa* ITRC5	2 mg/kg of soil	76% in 15 days	Soil	Kumar et al., 2011
	*Microbacterium* sp. ITRC1	200 mg/kg of soil	96% in 28 days	Soil	Manickam et al., 2006
	*Pseudoarthrobacter* sp.	5 mg/L	50.7% in 7 days	Soil	Nagpal and Paknikar, 2006
	*Pseudomonas* sp.	5 mg/L	52.2% in 7 days	Soil	Nagpal and Paknikar, 2006
	*Klebsiella* sp.	5 mg/L	51.2% in 7 days	Soil	Nagpal and Paknikar, 2006
	*Arthrobacter citreus* B1-100	100 mg/L	100% in 8 h	Soil	Datta et al., 2000
Fungi	*Ganoderma lucidum* GL-2	4 mg/L	75.5% in 28 days		Kaur and Kaur, 2016
	*Pleurotus ostreatus*	2.03 mg/L		Rotten wood	Dritsa and Rigas, 2013
	*Fusarium verticilliodes* AT-100	50 mg/L	30% in 7 days	Leaves	Guillén-Jiménez et al., 2012
	*Rhodotorula* sp. VITJzN03	600 mg/L	100% in 10 days	Soil	Salam et al., 2013
	*Fusarium poae*	100 mg/L	56.7% in 10 days	Soil	Sagar and Singh, 2011
	*Fusarium solani*	100 mg/L	59.4% in 10 days	Soil	Sagar and Singh, 2011
	*Conidiobolous* 03-1-56	5 mg/L	100% in 5 days	Soil	Nagpal et al., 2008
	*Bjendera audusta*	100 mg/kg of soil	69.1% in 30 days	Soil	Quintero et al., 2007
	*Cyathus bulleri*,	5 μM	97% in 28 days		Singh and Kuhad, 2000

Bacteria play a pivotal role in lindane biodegradation through chemical and physical interactions that lead to structural changes or complete degradation of the target molecule. Different bacteria were reported to degrade lindane at different rates. Singh and Singh (2019b) isolated and identified rhizospheric bacteria *Microbacterium* spp. strain P27 from *Phragmites karka*, which effectively degraded 82.7% lindane (50 mg⋅L^–1^) in 15 days and promoted plant growth as well. Sahoo et al. (2019) isolated *Paracoccus* sp. NITDBR1 from the agricultural fields of Manipur, which efficiently degraded 90% lindane (100 mg⋅L^–1^) in 8 days by using it as the sole source of carbon for its growth. Kumar (2018) isolated and characterized *Burkholderia* spp. strain IPL04 from a contaminated site by an enrichment culture method, which degraded 98% lindane in 8 days. The bacterial degradation of lindane has been extensively studied, however, only a few bacteria are known to completely mineralize lindane.

Fungal biodegradation is also considered an environment friendly approach for the detoxification of POPs in comparison with the traditional physical and chemical methods (Saez et al., 2018). Several studies reported the fungal degradation of lindane (Guillén-Jiménez et al., 2012; Salam et al., 2013; Kaur and Kaur, 2016). Fungi have extracellular enzymes that are relatively non-specific to the substrate and possess strong activity. These enzymes catalyze a wide range of reactions to break down lindane, and this might be induced by nutritional constraints (Nagpal et al., 2008).

The ligninolytic extracellular enzyme activity of white rot fungi efficiently degrades lindane. The soil environmental conditions (temperature, moisture content, and C/N ratio) and the presence of other microorganisms can affect the biodegradation activity of fungi (Kaur and Kaur, 2016). Kaur and Kaur (2016) reported 75.50% lindane degradation on the 28th day with the white rot fungus *Ganoderma lucidum* GL-2 strain grown in liquid-state fermentation. Guillén-Jiménez et al. (2012) isolated a non-white-rot fungus *Fusarium verticillioides* AT-100 from the leaves of *Agave tequilana* using an enrichment technique and then used lindane as the sole source of carbon and energy. This was the first report of lindane degradation by *F. verticillioides* to produce benzoic acid derivatives. *Rhodotorula* VITJzN03 was isolated from a sorghum cultivation field, and mineralized 100% lindane within 10 days at an initial concentration of 600 mg⋅L^–1^ (Salam et al., 2013).

## Microbial Metabolic Pathway of Lindane Degradation

Microbial metabolism can effectively degrade lindane through producing extracellular and intracellular enzymes that directly transform lindane into less toxic and non-toxic compounds (Alvarez et al., 2012). Lindane can be biodegraded under both aerobic and anaerobic conditions, however, it is generally mineralized only under aerobic conditions.

### Aerobic Degradation

**FIGURE 3:**
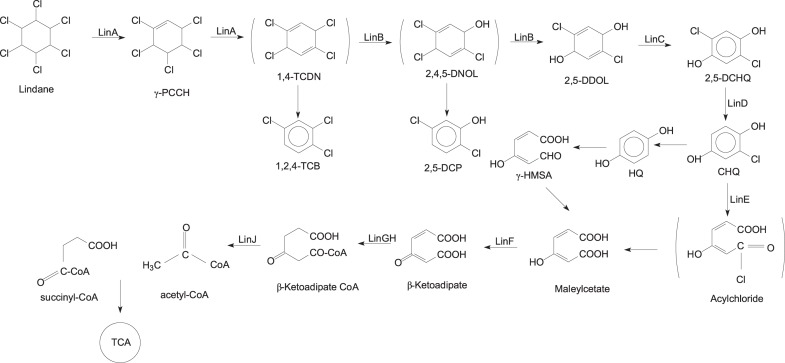
Aerobic degradation pathways of lindane.

Several aerobic microorganisms are known to degrade lindane, and the aerobic degradation pathway of lindane was extensively studied in *Sphingobium japonicum* UT26 (Nagata et al., 2007). UT26 uses lindane as a sole source of carbon and energy under aerobic conditions. Many studies found that the degradation pathways of other aerobic lindane degrading strains are the same or very similar to those in UT26. The main reactions that occur during microbial degradation of lindane include dehydrogenation, dechlorination, hydroxylation, dehydrochlorination, and mineralization (Cuozzo et al., 2017). The proposed aerobic biodegradation pathways of lindane are summarized in Figure 3. The key reaction during microbial degradation of lindane is the removal of the chlorine atom (Lal et al., 2006).

In this pathway, lindane is converted to 1,2,4-trichlorobenzene (1,2,4-TCB), 2,5-dichlorophenol (2,5-DCP), and 2,5-dichlorohydroquinone (2,5-DCHQ) by the enzymatic activities of dehydrochlorinase (LinA), halidohydrolase (LinB), and dehydrogenase (LinC). The degradation of lindane to 2,5-DCHQ is referred to as an upstream pathway, which is further metabolized through the downstream pathway (Endo et al., 2005). 2,5-DCHQ is converted to β-ketoadipate by reductive dechlorinase (LinD), ring-cleavage dioxygenase (LinE), and maleylacetate reductase (LinF). Researchers documented β-ketoadipate as a marker metabolite for the degradation of aromatic ring-containing compounds. The intermediate β-ketoadipate is further converted to succinyl-coenzyme A (CoA) and acetyl-CoA by succinyl-CoA: 3-oxoadipate CoA transferase (LinGH) and β-ketoadipyl CoA thiolase (LinJ). Both of these compounds are metabolized in the tricarboxylic acid (TCA) cycle (Nagata et al., 2007; Camacho-Pérez et al., 2012). In addition, other studies reported that lindane can be metabolized by microorganisms to produce pentachlorocyclohexene (PCCH), 3,4,5,6-tetrachloro-1-cyclohexene (TCCH), pentachlorobenzene (PCB), or trichlorobenzene (TCB) (Geueke et al., 2013; Cuozzo et al., 2017).

### Anaerobic Degradation

**FIGURE 4:**
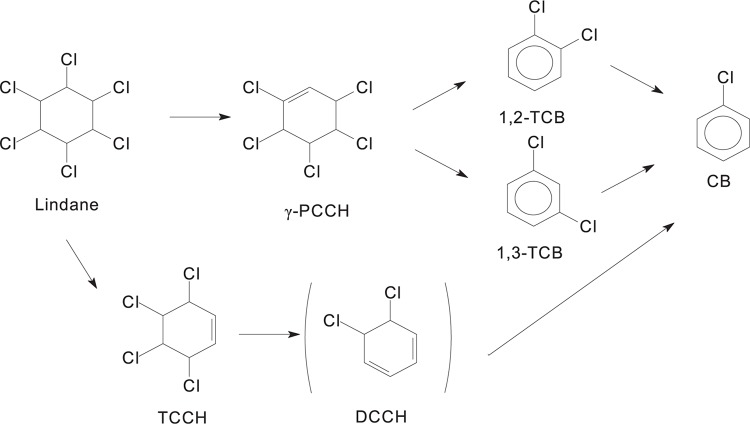
Anaerobic degradation pathways of lindane.

Several studies reported that the microbial anaerobic degradation of lindane is different from aerobic degradation (Endo et al., 2005; Bashir et al., 2018). Dichloro-elimination and dehydrochlorination appear to be the main processes during the transformation of lindane via tetrachlorocyclohexene (TCCH) into lower chlorinated products under the anoxic conditions of aquifer sediments (Lal et al., 2010). There are two different descriptions of the microbial anaerobic degradation pathways of lindane (Figure 4). Some studies proposed that the degradation proceeds through two dichloroeliminations, resulting in the formation of 3,4,5,6-tetrachloro-1-cyclohexene (γ-TCCH) followed by 5,6-dichlorocyclohexa-1,2-diene. Chlorobenzene is then produced by a dehydrochlorination reaction (Saez et al., 2017). Another anaerobic degradation pathway produces pentachlorocyclohexene (PCCH) followed by 1,2-dichlorobenzene (1,2-DCB) and 1,3-dichlorobenzene (1,3-DCB) (Quintero et al., 2005a, b). Anaerobic bacteria from the sediments and pure cultures of sulfate-reducing bacteria influenced lindane dehalogenation resulting in the accumulation of final products, such as monochlorobenzene and benzene (Camacho-Pérez et al., 2012).

## Functional Enzymes Involved in Lindane Degradation

Dehalogenases are the key enzymes that degrade various halogenated compounds through cleavage of the carbon chlorine stable bond (Lal et al., 2010). Lindane has six chlorine atoms per molecule and dechlorination is a significant step in its degradation. As shown in Figure 3, the biodegradation of lindane requires the participation of various enzymes. The dehalogenases that degrade lindane are dehydrochlorinase (LinA), dehalogenase (LinB), and reductive dechlorinase (LinD) (Nagata et al., 2007). Dehydrogenase (LinC), ring cleavage oxygenase (LinE), and maleylacetate reductase (LinF), which catalyze the dechlorination of substrates, can also be generally categorized as dehalogenases (Lal et al., 2006).

In general, LinA, LinB, and LinC are upstream pathway enzymes of lindane degradation. LinA is a homotetrameric protein with a molecular mass of 16.5 kDa (Nanasato et al., 2016). LinA belongs to the dehydrohalogenase class of enzymes, which can eliminate HCl from a substrate molecule to form a double bond (Cuozzo et al., 2017). LinA mediates the first two dehydrochlorination steps of lindane (Manickam et al., 2008). Additionally, LinA does not require cofactors to catalyze its activity and thus is a unique dehydrogenase enzyme that distinguishes between the two previously reported dehydrochlorinases (dichlorodiphenyltrichloroethane (DDT) dehydrochlorinase and 3-chloro-D-alanine dehydrochlorinase).

Unlike LinA, LinB is a monomeric 32 kDa protein located in the periplasm of sphingomonads (Geueke et al., 2013). LinB is a haloalkane dehalogenase of the α/β-hydrolase family and possesses relatively broad substrate specificity (Janssen, 2004). LinB is a key enzyme for lindane degradation in soil and catalyzes dehalogenation through a hydrolytic mechanism. LinB is responsible for the hydrolytic dechlorination of 1,3,4,6-tetrachloro-1,4-cyclohexadiene (1,4-TCDN) to 2,5-dichloro-2,5-cyclohexadiene-1,4-diol (2,5-DDOL) during the aerobic degradation pathway of lindane (Jan et al., 2005). LinB was found to be involved in the degradation of β- and δ-HCH up to different levels.

Less is currently known about the third upstream pathway enzyme LinC or any other downstream pathway proteins. LinC (28 kDa) is considered a 2,5-DDOL dehydrogenase in the short-chain alcohol dehydrogenase family (Rinku et al., 2005). A general catalytic mechanism explained that hydride transfer from the substrate to NAD^–^ forms NADPH and a reduced product [the conversion of 2,5-DDOL to 2,5-dichlorohydroquinone (2,5-DCHQ)]. LinD (38.4 kDa), LinE (36.0 kDa), LinF (38.0 kDa), and LinR (33.6 kDa) (a transcriptional regulator) are associated with the downstream lindane degradation pathway (Lal et al., 2010). In *S. japonicum* UT26, LinD and LinE were induced by LinR in the presence of 2,5-DCHQ, chlorohydroquinone (CHQ) and hydroquinone (HQ) (Keisuke et al., 2002). As the degradation of lindane can lead to different intermediate compounds, we must also consider key enzymes for the further degradation of commonly reported lindane metabolites. To further understand the mechanisms of lindane biodegradation, it is necessary to explore further key enzymes of lindane degradation.

## Bioremediation of Lindane-Contaminated Environments

Lindane is an organochlorine pesticide that is highly persistent in the environment due to frequent crop spraying, field applications, accidental spills, domestic wastes, industrial effluents, and garbage dumping (Singh and Singh, 2019b). Lindane residues permeate through the soil surface to the groundwater and cause the extensive pollution of aquatic ecosystems. Lindane accumulation has been reported worldwide and is very toxic to the environment, humans, and animals (Pamela et al., 2011). Therefore, urgent consideration and management of the problem is necessary. Bioremediation is the most promising biotechnological approach to clean polluted environments (Egorova et al., 2017; Zhang J. et al., 2018; Bhatt et al., 2020a). The bacterial strains isolated from sponges demonstrate robustness in lindane degradation. Bacteria associated with the sponge *Hymeniacidon perlevis* were found to be suitable for lindane degradation. The bacteria isolated belong to *Mameliella phaeodactyli*, *Pseudovibrio ascidiaceicola*, *Oceanicaulis stylophorae*, *Ruegeria atlantica*, and other uncharacterized genera (Loredana et al., 2017). For lindane bioremediation in samples collected from different polluted sites, researchers applied novel techniques (Asemoloye et al., 2017; Salam et al., 2017; Wang et al., 2019). Pure cultures, microbial consortium, nanobiotechnology, and plant–microbe interactions are discussed below along with their successful outcomes.

### Pure Cultures

Considering the vast amount of lindane used and the residues in the environment, attempts were made in the past to use pure cultures for the decontamination of lindane. As previously described, a large number of microorganisms with lindane degradation capacity were isolated and characterized (Table 1). Several studies described the application of a single microorganism for the bioremediation of lindane-contaminated soil (Aparicio et al., 2015; Salam et al., 2017). *Streptomyces* sp. M7 exhibited strong versatility, showing the ability to bioremediate lindane contaminated soil samples at several physicochemical conditions (Aparicio et al., 2015).

The ascomycetous yeast strain *Candida* VITJzN04 has the ability to degrade 78% of lindane in garden soils (lindane ∼100 mg/kg) within 30 days (Salam et al., 2017). The traditional microbial methods are based on the culture dependent approach (Zhan et al., 2018a; Bhatt et al., 2020b). However, the ability of a single microorganism to degrade lindane is influenced not only by its genetic properties but also by the environmental conditions, such as the pH, temperature, concentration of lindane, etc. (Feng et al., 2020).

### Microbial Consortium

A microbial consortium performs better than axenic cultures due to robustness in metabolic function (Bhatt et al., 2019b). A mixed community of microorganisms can alleviate the metabolic limitations of a single population (Shong et al., 2012). In nature, microorganisms coexist as part of microbial consortia where multiple populations carry out complex chemical and physiological functions for the survival of the community (Polti et al., 2014). Natural and artificial microbial consortia are of great significance for the degradation and removal of lindane. Murthy and Manonmani (2007) developed a microbial consortium against lindane and optimized the degradation conditions. The consortium rapidly degraded all concentrations of lindane up to 25 mg/L.

Saez et al. (2015) evaluated the influence of a *Streptomyces* consortium on lindane degradation in liquid and slurry systems. An actinobacteria consortium was stable and removed 97% of the lindane. Aparicio et al. (2017) established the optimal biological and physicochemical parameters to simultaneously remove lindane and Cr (VI) from the soil with an actinobacteria consortium of *Streptomyces* sp. M7, MC1, and A5 and *Amycolatopsis tucumanensis* AB0. An actinobacteria consortium was found to be superior for the degradation of lindane from a contaminated environment.

Kumar et al. (2017) conducted a bath culture degradation analysis and reported the enhanced codegradation of chlorpyrifos and lindane with two or more strains. Elcey and Kunhi (2009) isolated a lindane-degrading consortium from a sugarcane field. The consortium mineralized 300 μg/mL of lindane after 108 h of acclimation in the presence of a substrate, and no apparent accumulation of intermediary metabolites was observed.

Microbial consortiums communicated with each other via the mechanism known as quorum sensing (Ye et al., 2019, 2020). Due to this communication in the lindane-contaminated sites, the metabolic burden is divided into various members and the consortium perform complex functions. In nature, microbes coexists as communities that are affected by the local environmental conditions. Mixed microbial cultures may compete for the same resources in nature, however, they can cross feed with each other and live symbiotically with a single carbon source (Mas et al., 2016). The pesticides are degraded by the microbial consortium, which shares the metabolic labor during the degradation (Billet et al., 2019). A consortium of *Streptomyces* was able to efficiently degrade various chlorinated pesticides (Fuentes et al., 2013). A bacterial consortium (10 bacterial strains) was able to simultaneously degrade organophosphorous and organochlorine pesticides within 24 h (Abraham et al., 2014).

### Nanobiotechnology

Currently, an integrated nanobiotechnological approach for the treatment of pesticides is gaining popularity as a novel and effective technology. Over the past decade, studies investigated the combination of nanoscale inorganic particles and microbial cells in the degradation of chlorinated hydrocarbons (Wang and Tseng, 2009). These studies proposed reductive dechlorination of lindane by nanoparticles followed by oxidative degradation of metabolites by the microbial cells. Salam and Das (2015) evaluated the effect of an embedded bio-nano hybrid system using nanoscale zinc oxide (n-ZnO) and the lindane-degrading yeast *Candida* VITJzN04 for lindane degradation. The bio-nano hybrid degraded lindane more effectively than the native yeasts and completely removed lindane within 3 days.

Singh et al. (2013) used an integrated nano biotechnique to combine stabilized Pd/Fe (0) bimetallic nanoparticles [carboxymethyl cellulose (CMC)-Pd/Fe (0)] with *Sphingomonas* spp. strain NM05 for lindane degradation in contaminated soil. Their study signifies the potential efficacy of an integrated technique as an effective alternative remedial tool for lindane-contaminated soils. The embedded bio-nano hybrid system can be applied as an effective remediation method for the treatment of lindane-contaminated soils and wastewaters.

### Plant–Microbe Association

Plants also possess a remarkable ability to remove or immobilize various environmental pollutants (Asemoloye et al., 2017). A unique chemical communication occurs between plants and their rhizospheric microbes that can be used to repair the polluted environment. In the plant–microbe relationship, plants provide habitats and nutrients for their associated bacteria. These bacteria can provide the plant with improved stress resistance, growth, and degradation of environmental pollutants (Singh and Singh, 2019b). Therefore, plant–microbe-associated bioremediation techniques were reported as cost-efficient and eco-friendly methods of cleaning polluted sites.

Sainz et al. (2010) investigated the effects of lindane pollution on vegetables and the associated arbuscular mycorrhiza. Their research indicated that the fungus increases the plant’s tolerance toward the toxic effects of soil conditions. Becerra-Castro et al. (2013) inoculated substrates seeded with *Cytisus striatus*, *Rhodococcus erythropolis* ET54b, and *Sphingomonas* sp. D4. The results showed that the inoculation of *C. striatus* with a combination of bacterial strains is a promising approach for the remediation of lindane-contaminated sites. Asemoloye et al. (2017) isolated five dominant fungal strains from an aged lindane polluted site. Lindane degradation in polluted soil was reported to be more effective in response to the combined actions of plant roots and fungi. However, the plant–microbe association bioremediation technique for lindane degradation is still in the primary stages. There is a need for large-scale and in-depth evaluation of bioremediation protocols, as they may be useful for the remediation of lindane-polluted soils.

### Novel Technologies

Traditional technologies, such as pure cultures, lack widespread application due to the low remediation capacity and sensitivity to environmental conditions. Therefore, based on the limitations of traditional technology and the modern molecular biology technology, some novel technologies that are considered to be highly efficient, eco-friendly, and promising tools were proposed to remediate the contaminated sites, for instance, metagenomics, genetic engineering, and enzyme engineering. With the development of new techniques, including metagenomics and genetic engineering, it becomes easy to explore new microorganisms and genes/enzymes, and these can be deployed in bioremediation programs (Kumar and Pannu, 2018).

One novel approach to enzyme immobilization is based on the entrapment of magnetic nanoparticles via epoxy cross-linking. This technique can be employed to improve the activity of pollutant degradation bacteria, fungi, or heterologous-expressed enzymes (Iype et al., 2017; Gangola et al., 2018). Recent high throughput techniques can be used to explore key functional strains from the pesticide degradation microbial community and fulfill the gap between laboratory cultures and large-scale applications (Chen et al., 2019).

Omics-based technologies provided insights into pesticide degradation (Bhatt, 2019). Advancements in next generation sequencing provide a huge amount of microbial data in the form of nucleotides and protein sequences that are deposited in various online databases (NCBI, DDBJ, Uniprot, PDB, etc.) (Bhatt and Barh, 2018). The data from the omics-based approaches can be used for the identification of novel genes and enzymes and molecular docking studies (Bhatt, 2018). These databases are also helpful for the classification of novel lindane-degrading microbes and phylogenetic analysis.

Microbial systems biology is another recent tool to study the lindane-degrading microbes and their physiology in a contaminated environment (Bhatt, 2019). The systems biology of pesticide-degrading microbial cells is helpful for understanding the detailed metabolic pathways, lindane residues in the soil/water system and their effect on other living systems. Using this tool, we can analyze the overall biomagnification due to lindane in the environment (Bhatt et al., 2019b). Systems biology tools are also helpful to design the synthetic microbial consortium for the rapid removal of lindane from soil and water bodies. With the development of modern science and technology, new technologies will continue to be developed and applied for *in situ* and *ex situ* remediation-based environmental management programs.

## Conclusion and Future Perspectives

Lindane, a persistent organochlorine pollutant, has become a major environmental problem around the world. Biodegradation based on the catabolic activity of pesticide-degrading microorganisms has emerged as the most cost effective, eco-friendly, and promising strategy to eliminate environmental lindane residues. The research mainly focused on the screening of lindane-degrading strains and the analysis of degradation products under laboratory conditions. A few studies reported the degradation mechanisms and practical applications of specific strains. Given the health hazards and the environmental impact of lindane, the microbial degradation-based bioremediation methods and associated technologies should be widely developed and applied. Increasing the information available regarding degradation pathways with regulatory genes and enzymes will help with the development of novel remediation technologies. Moreover, advanced molecular approaches, enzyme engineering, and emerging materials will provide better tools for the remediation of harmful pollutants.

## Author Contributions

SC conceived the presented idea. WZ contributed to the writing and prepared figures and table. ZL, SP, PB, and SC participated in revising the manuscript. All authors approved it for publication.

## Conflict of Interest

The authors declare that the research was conducted in the absence of any commercial or financial relationships that could be construed as a potential conflict of interest.

## References

[B1] Abhilash P. Singh N. (2010). *Withania somnifera* Dunal-mediated dissipation of lindane from simulated soil: implications for rhizoremediation of contaminated soil. *J. Soils Sediments* 10 272–282. 10.1007/s11368-009-0085-x

[B2] Abolhassani M. Asadikaram G. Paydar P. Fallah H. Aghaee-Afshar M. Moazed V. (2019). Organochlorine and organophosphorous pesticides may induce colorectal cancer; a case-control study. *Ecotoxicol. Environ. Saf.* 178 168–177. 10.1016/j.ecoenv.2019.04.030 31004929

[B3] Abraham J. Silambarasan S. Logeswari P. (2014). Simultaneous degradation of organophosphorus and organochlorine pesticides by bacterial consortium. *J. Taiwan Inst. Chem. Eng.* 45 2590–2596. 10.1016/j.jtice.2014.06.014

[B4] Agrahari A. Singh A. Srivastava A. Jha R. R. Patel D. K. Yadav S. (2019). Overexpression of cerebral cytochrome P450s in prenatally exposed offspring modify the toxicity of lindane in rechallenged offspring. *Toxicol. Appl. Pharmacol.* 371 20–37. 10.1016/j.taap.2019.03.022 30926376

[B5] Alvarez A. Benimeli C. Saez J. Fuentes M. Cuozzo S. Polti M. (2012). Bacterial bio-resources for remediation of hexachlorocyclohexane. *Int. J. Mol. Sci.* 13 15086–15106. 10.3390/ijms131115086 23203113 PMC3509629

[B6] Anupama K. Paul S. (2009). Ex situ and in situ biodegradation of lindane by Azotobacter chroococcum. *J. Environ. Sci. Health Part B* 45 58–66. 10.1080/03601230903404465 20390932

[B7] Aparicio J. Solá M. Z. S. Benimeli C. S. Amoroso M. J. Polti M. A. (2015). Versatility of *Streptomyces* sp. M7 to bioremediate soils co-contaminated with Cr (VI) and lindane. *Ecotoxicol. Environ. Saf.* 116 34–39. 10.1016/j.ecoenv.2015.02.036 25749405

[B8] Aparicio J. D. Benimeli C. S. Almeida C. A. Polti M. A. Colin V. L. (2017). Integral use of sugarcane vinasse for biomass production of actinobacteria: potential application in soil remediation. *Chemosphere* 181 478–484. 10.1016/j.chemosphere.2017.04.107 28460294

[B9] Arora P. K. Srivastava A. Garg S. K. Singh V. P. (2017). Recent advances in degradation of chloronitrophenols. *Bioresour. Technol.* 250 902–909. 10.1016/j.biortech.2017.12.007 29229201

[B10] Asemoloye M. D. Ahmad R. Jonathan S. G. (2017). Synergistic rhizosphere degradation of γ-hexachlorocyclohexane (lindane) through the combinatorial plant-fungal action. *PLoS One* 12:e0183373. 10.1371/journal.pone.0183373 28859100 PMC5578508

[B11] Bajaj S. Sagar S. Khare S. Singh D. K. (2017). Biodegradation of γ-hexachlorocyclohexane (lindane) by halophilic bacterium *Chromohalobacter* sp. LD2 isolated from HCH dumpsite. *Int. Biodeterior. Biodegrad.* 122 23–28. 10.1016/j.ibiod.2017.04.014

[B12] Bashir S. Kuntze K. Vogt C. Nijenhuis I. (2018). Anaerobic biotransformation of hexachlorocyclohexane isomers by *Dehalococcoides* species and an enrichment culture. *Biodegradation* 29 409–418. 10.1007/s10532-018-9838-9 29916096

[B13] Becerra-Castro C. Kidd P. S. Rodríguez-Garrido B. Monterroso C. Santos-Ucha P. Prieto-Fernández Á (2013). Phytoremediation of hexachlorocyclohexane (HCH)-contaminated soils using *Cytisus striatus* and bacterial inoculants in soils with distinct organic matter content. *Environ. Pollut.* 178 202–210. 10.1016/j.envpol.2013.03.027 23583940

[B14] Benimeli C. Fuentes M. Abate C. Amoroso M. (2008). Bioremediation of lindane-contaminated soil by *Streptomyces* sp. M7 and its effects on *Zea mays* growth. *Int. Biodeterior. Biodegrad.* 61 233–239. 10.1016/j.ibiod.2007.09.001

[B15] Beyer A. Matthies M. (2001). Long-range transport potential of semivolatile organic chemicals in coupled air-water systems. *Environ. Sci. Pollut. Res.* 8 173–179. 10.1007/BF02987382 11505901

[B16] Bhatt P. (2018). “Insilico tools to study the bioremediation in microorganisms,” in *Handbook of Research on Microbial Tools for Environmental Waste Management*, eds Pathak V. Navneet (Hershey, PA: IGI Global), 389–395. 10.4018/978-1-5225-3540-9.ch018

[B17] Bhatt P. (2019). *Smart Bioremediation Technologies: Microbial Enzymes.* Amsterdam: Elsevier Science 10.4018/978-1-5225-3540-9.ch018

[B18] Bhatt P. Barh A. (2018). “Bioinformatic tools to study the soil microorganisms: an in silico approach for sustainable agriculture,” in *In Silico Approach for Sustainable Agriculture*, eds Choudhary D. Kumar M. Prasad R. Kumar V., (Singapore: Springer).

[B19] Bhatt P. Bhatt K. Huang Y. Lin Z. Chen S. (2020a). Esterase is a powerful tool for the biodegradation of pyrethroid insecticides. *Chemosphere* 244:125507. 10.1016/j.chemosphere.2019.125507 31835049

[B20] Bhatt P. Huang Y. Rene E. R. Kumar A. J. Chen S. (2020b). Mechanism of allethrin biodegradation by a newly isolated Sphingomonas trueperi strain CW3 from wastewater sludge. *Bioresour. Technol.* 305:123074. 10.1016/j.biortech.2020.123074 32146283

[B21] Bhatt P. Huang Y. Zhan H. Chen S. (2019a). Insight into microbial applications for the biodegradation of pyrethroid insecticides. *Front. Microbiol.* 10:1778. 10.3389/fmicb.2019.01778 31428072 PMC6687851

[B22] Bhatt P. Huang Y. Zhang W. Sharma A. Chen S. (2020c). Enhanced cypermethrin degradation kinetics and metabolic pathway in Bacillus thuringiensis strain SG4. *Microorganisms* 8:223. 10.3390/microorganisms8020223 32046050 PMC7074683

[B23] Bhatt P. Pal K. Bhandari G. Barh A. (2019b). Modelling of the methyl halide biodegradation on bacteria and its effect on other environmental systems. *Pest. Biochem. Physiol.* 158 88–100. 10.1016/j.pestbp.2019.04.015 31378365

[B24] Billet L. Devers M. Rouard N. Laurent F. M. Spor A. (2019). Labour sharing promotes coexistence in atrazine degrading bacterial communities. *Sci. Rep.* 9:18363. 10.1038/s41598-019-54978-2 31798012 PMC6892810

[B25] Camacho-Pérez B. Ríos-Leal E. Rinderknecht-Seijas N. Poggi-Varaldo H. M. (2012). Enzymes involved in the biodegradation of hexachlorocyclohexane: a mini review. *J. Environ. Manage.* 95 306–318. 10.1016/j.jenvman.2011.06.047 21992990

[B26] Chen S. Chang C. Deng Y. An S. Dong Y. Zhou J. (2014). Fenpropathrin biodegradation pathway in *Bacillus* sp. DG-02 and its potential for bioremediation of pyrethroid-contaminated soils. *J. Agric. Food. Chem.* 62 2147–2157. 10.1021/jf404908j 24576059

[B27] Chen S. Deng Y. Chang C. Lee J. Cheng Y. Cui Z. (2015). Pathway and kinetics of cyhalothrin biodegradation by Bacillus thuringiensis strain ZS-19. *Sci. Rep.* 5:8784. 10.1038/srep08784 25740758 PMC4350101

[B28] Chen S. Dong Y. H. Chang C. Deng Y. Xi F. Z. Zhong G. (2013). Characterization of a novel cyfluthrin-degrading bacterial strain *Brevibacterium aureum* and its biochemical degradation pathway. *Bioresour. Technol.* 132 16–23. 10.1016/j.biortech.2013.01.002 23395753

[B29] Chen S. Geng P. Xiao Y. Hu M. (2012). Bioremediation of β-cypermethrin and 3-phenoxybenzaldehyde contaminated soils using *Streptomyces aureus* HP-S-01. *Appl. Microbiol. Biotechnol.* 94 505–515. 10.1007/s00253-011-3640-5 22038248

[B30] Chen S. Hu M. Liu J. Zhong G. Yang L. Rizwan-ul-Haq M. (2011). Biodegradation of beta-cypermethrin and 3-phenoxybenzoic acid by a novel *Ochrobactrum lupini* DG-S-01. *J. Hazard. Mater.* 187 433–440. 10.1016/j.jhazmat.2011.01.049 21282001

[B31] Chen Y. Zhang L. Feng L. Chen G. Wang Y. Zhai Z. (2019). Exploration of the key functional strains from an azo dye degradation microbial community by DGGE and high-throughput sequencing technology. *Environ. Sci. Pollut. Res. Int.* 6 24658–24671. 10.1007/s11356-019-05781-z 31236867

[B32] Cuozzo S. A. Sineli P. E. Davila Costa J. Tortella G. (2017). *Streptomyces* sp. is a powerful biotechnological tool for the biodegradation of HCH isomers: biochemical and molecular basis. *Crit. Rev. Biotechnol.* 38 719–728. 10.1080/07388551.2017.1398133 29124958

[B33] Cycoń M. Mrozik A. Piotrowska-Seget Z. (2017). Bioaugmentation as a strategy for the remediation of pesticide-polluted soil: a review. *Chemosphere* 172 52–71. 10.1016/j.chemosphere.2016.12.129 28061345

[B34] Datta J. Maiti A. K. Modak D. P. Chakrabartty P. K. Bhattacharyya P. Ray P. K. (2000). Metabolism of gamma-hexachlorocyclohexane by *Arthrobacter citreus* strain BI-100: Identification of metabolites. *J. Gen. Appl. Microbiol.* 46 59–67. 10.2323/jgam.46.59 12483592

[B35] De Paolis M. Lippi D. Guerriero E. Polcaro C. Donati E. (2013). Biodegradation of α-, β-, and γ-hexachlorocyclohexane by *Arthrobacter fluorescens* and *Arthrobacter giacomelloi*. *Appl. Biochem. Biotechnol.* 170 514–524. 10.1007/s12010-013-0147-9 23553101

[B36] Dominguez C. M. Oturan N. Romero A. Santos A. Oturan M. A. (2018a). Lindane degradation by electrooxidation process: effect of electrode materials on oxidation and mineralization kinetics. *Water Res.* 135 220–230. 10.1016/j.watres.2018.02.037 29477060

[B37] Dominguez C. M. Oturan N. Romero A. Santos A. Oturan M. A. (2018b). Removal of lindane wastes by advanced electrochemical oxidation. *Chemosphere* 202 400–409. 10.1016/j.chemosphere.2018.03.124 29579675

[B38] Dritsa V. Rigas F. (2013). The ligninolytic and biodegradation potential on lindane of *Pleurotus ostreatus* spp. *J. Min. World Express* 2 23–30.

[B39] Egorova D. O. Buzmakov S. A. Nazarova E. A. Andreev D. N. Demakov V. A. Plotnikova E. G. (2017). Bioremediation of hexachlorocyclohexane -contaminated soil by the new Rhodococcus wratislaviensis Strain Ch628. *Water Air Soil Pollut.* 228:183 10.1007/s11270-017-3344-2

[B40] Elcey C. D. Kunhi A. M. (2009). Substantially enhanced degradation of hexachlorocyclohexane isomers by a microbial consortium on acclimation. *J. Agric. Food Chem.* 58 1046–1054. 10.1021/jf9038259 20041660

[B41] Endo R. Kamakura M. Miyauchi K. Fukuda M. Ohtsubo Y. Tsuda M. (2005). Identification and characterization of genes involved in the downstream degradation pathway of γ-hexachlorocyclohexane in *Sphingomonas paucimobilis* UT26. *J. Bacteriol.* 187 847–853. 10.1128/JB.187.3.847-853.2005 15659662 PMC545726

[B42] Feng Y. Huang Y. Zhan H. Bhatt H. Chen S. (2020). An overview of strobilurin fungicide degradation: current status and future perspective. *Front. Microbiol.* 10:389 10.3389/fmicb.2020.00389 PMC7081128 32226423

[B43] Fuentes M. S. Briceno G. E. Saez J. M. Benimeli C. S. Diez M. C. Amorosso M. Z. (2013). Enhanced removal of a pesticides mixture by single cultures and consortia of free and immobilized *Streptomyces* strains. *Biomed Res. Int.* 2013:392573. 10.1155/2013/392573 23865051 PMC3705853

[B44] Gangola S. Sharma A. Bhatt P. Khati P. Chaudhary P. (2018). Presence of esterase and laccase in *Bacillus subtilis* facilitates biodegradation and detoxification of cypermethrin. *Sci. Rep.* 8:12755. 10.1038/s41598-018-31082-5 30143738 PMC6109164

[B45] Geueke B. Garg N. Ghosh S. Fleischmann T. Holliger C. Lal R. (2013). Metabolomics of hexachlorocyclohexane (HCH) transformation: ratio of LinA to LinB determines metabolic fate of HCH isomers. *Environ. Microbiol.* 15 1040–1049. 10.1111/1462-2920.12009 23121161

[B46] Giri K. Rawat A. P. Rawat M. Rai J. (2014). Biodegradation of hexachlorocyclohexane by two species of bacillus isolated from contaminated soil. *Chem. Ecol.* 30 97–109. 10.1080/02757540.2013.844795 23322487

[B47] Githinji I. N. (2015). *Screening and Isolation of γ-Hexachlorocyclohexane Degrading Bacteria from Contaminated Soil in Kenya.* Nairobi: University of Nairobi 10.1080/02757540.2013.844795

[B48] Guillén-Jiménez F. D. M. Cristiani-Urbina E. Cancino-Díaz J. C. Flores-Moreno J. L. Barragán-Huerta B. E. (2012). Lindane biodegradation by the Fusarium verticillioides AT-100 strain, isolated from *Agave tequilana* leaves: kinetic study and identification of metabolites. *Int. Biodeterior. Biodegrad.* 74 36–47. 10.1016/j.ibiod.2012.04.020

[B49] Huang Y. Zhan H. Bhatt P. Chen S. (2019). Paraquat degradation from contaminated environments: current achievements and perspectives. *Front. Microbiol.* 10:1754. 10.3389/fmicb.2019.01754 31428067 PMC6689968

[B50] Iype T. Thomas J. Mohan S. Johnson K. K. George L. E. Ambattu L. A. (2017). A novel method for immobilization of proteins via entrapment of magnetic nanoparticles through epoxy cross-linking. *Anal. Biochem.* 519 42–50. 10.1016/j.ab.2016.12.007 27965063

[B51] Jan K. Kamila H. Tomás J. Yuji N. Ana N. Federico G. (2005). Quantitative analysis of substrate specificity of haloalkane dehalogenase LinB from *Sphingomonas paucimobilis* UT26. *Biochemistry* 44 3390–3401. 10.1021/bi047912o 15736949

[B52] Janssen D. B. (2004). Evolving haloalkane dehalogenases. *Curr. Opin. Chem. Biol.* 8 150–159. 10.1016/j.cbpa.2004.02.012 15062775

[B53] Jayaraj R. Megha P. Sreedev P. (2016). Organochlorine pesticides, their toxic effects on living organisms and their fate in the environment. *Interdiscip. Toxicol.* 9 90–100. 10.1515/intox-2016-0012 28652852 PMC5464684

[B54] Jung H. J. Koutavarapu R. Lee S. Kim J. H. Choi H. C. Choi M. Y. (2018). Enhanced photocatalytic degradation of lindane using metal–semiconductor Zn@ ZnO and ZnO/Ag nanostructures. *J. Environ. Sci.* 74 107–115. 10.1016/j.jes.2018.02.014 30340663

[B55] Kaur H. Kaur G. (2016). Application of ligninolytic potentials of a white-rot fungus Ganoderma lucidum for degradation of lindane. *Environ. Monit. Assess.* 188:588. 10.1007/s10661-016-5606-7 27670886

[B56] Keisuke M. Haeng-Seog L. Masao F. Masamichi T. Yuji N. (2002). Cloning and characterization of linR, involved in regulation of the downstream pathway for gamma-hexachlorocyclohexane degradation in *Sphingomonas paucimobilis* UT26. *Mol. Immunol.* 68 1803–1807. 10.1128/AEM.68.4.1803-1807.2002 11916699 PMC123885

[B57] Kumar D. (2018). Biodegradation of γ-Hexachlorocyclohexane by *Burkholderia* sp. IPL04. *Biocatal. Agric. Biotechnol.* 16 331–339. 10.1016/j.bcab.2018.09.001

[B58] Kumar D. Jaswal S. Chopra S. (2017). Co-degradation study of lindane and chlorpyrifos by novel bacteria. *Int. J. Environ. Waste Manag.* 20 283–299. 10.1504/IJEWM.2017.090050

[B59] Kumar D. Kumar A. Sharma J. (2016). Degradation study of lindane by novel strains *Kocuria* sp. DAB-1Y and *Staphylococcus* sp. DAB-1W. *Bioresour. Bioprocess.* 3:53. 10.1186/s40643-016-0130-8 28090433 PMC5196013

[B60] Kumar D. Pannu R. (2018). Perspectives of lindane (γ-hexachlorocyclohexane) biodegradation from the environment: a review. *Bioresour. Bioprocess.* 5:29 10.1186/s40643-018-0213-9

[B61] Kumar M. Gupta S. K. Garg S. K. Kumar A. (2011). Biodegradation of hexachlorocyclohexane-isomers in contaminated soils. *Soil Biol. Biochem.* 38 2318–2327. 10.1016/j.soilbio.2006.02.010

[B62] Lal R. Dogra C. Malhotra S. Sharma P. Pal R. (2006). Diversity, distribution and divergence of lin genes in hexachlorocyclohexane-degrading sphingomonads. *Trends Biotechnol.* 24 121–130. 10.1016/j.tibtech.2006.01.005 16473421

[B63] Lal R. Pandey G. Sharma P. Kumari K. Malhotra S. Pandey R. (2010). Biochemistry of microbial degradation of hexachlorocyclohexane and prospects for bioremediation. *Microbiol. Mol. Biol. Rev.* 74 58–80. 10.1128/MMBR.00029-09 20197499 PMC2832351

[B64] Lin Z. Zhang W. Pang S. Huang Y. Mishra S. Bhatt P. (2020). Current approaches to and future perspectives on methomyl degradation in contaminated soil/water environments. *Molecules* 25:738. 10.3390/molecules25030738 32046287 PMC7036768

[B65] Liu J. Chen S. Ding J. Xiao Y. Han H. Zhong G. (2015). Sugarcane bagasse as support for immobilization of *Bacillus pumilus* HZ-2 and its use in bioremediation of mesotrione-contaminated soils. *Appl. Microbiol. Biotechnol.* 99 10839–10851. 10.1007/s00253-015-6935-0 26337896

[B66] Loredana S. Graziano P. Antonio M. Carlotta N. M. Caterina L. Maria A. A. (2017). Lindane bioremediation capability of bacteria associated with the demosponge *Hymeniacidon perlevis*. *Mar. Drugs* 15:E108. 10.3390/md15040108 28383507 PMC5408254

[B67] Luo D. Pu Y. Tian H. Cheng J. Zhou T. Tao Y. (2016). Concentrations of organochlorine pesticides in umbilical cord blood and related lifestyle and dietary intake factors among pregnant women of the Huaihe River Basin in China. *Environ. Int.* 92 276–283. 10.1016/j.envint.2016.04.017 27123771

[B68] Manickam N. Mau M. Schlömann M. (2006). Characterization of the novel HCH-degrading strain, *Microbacterium* sp. ITRC1. *Appl. Microbiol. Biotechnol.* 69 580–588. 10.1007/s00253-005-0162-z 16315057

[B69] Manickam N. Misra R. Mayilraj S. (2010). A novel pathway for the biodegradation of gamma-hexachlorocyclohexane by a *Xanthomonas* sp. strain ICH12. *J. Appl. Microbiol.* 102 1468–1478. 10.1111/j.1365-2672.2006.03209.x 17578411

[B70] Manickam N. Reddy M. Saini H. Shanker R. (2008). Isolation of hexachlorocyclohexane-degrading *Sphingomonas* sp. by dehalogenase assay and characterization of genes involved in γ-HCH degradation. *J. Appl. Microbiol.* 104 952–960. 10.1111/j.1365-2672.2007.03610.x 18042212

[B71] Markman S. Guschina I. A. Barnsley S. Buchanan K. L. Pascoe D. Müller C. T. (2007). Endocrine disrupting chemicals accumulate in earthworms exposed to sewage effluent. *Chemosphere* 70 119–125. 10.1016/j.chemosphere.2007.06.045 17675209

[B72] Mas A. Jamshidi S. Lagadeuc Y. Eveillard D. Vandenkoornhuyse P. (2016). Beyond the black queen hypothesis. *ISME J.* 10 2085–2093. 10.1038/ismej.2016.22 26953598 PMC4989313

[B73] Mladenović D. Djuric D. Petronijević N. Radosavljević T. Radonjić N. Matić D. (2010). The correlation between lipid peroxidation in different brain regions and the severity of lindane-induced seizures in rats. *Mol. Cell. Biochem.* 333:243. 10.1007/s11010-009-0225-z 19693653

[B74] Mortazavi N. Asadikaram G. Ebadzadeh M. R. Kamalati A. Pakmanesh H. Dadgar R. (2019). Organochlorine and organophosphorus pesticides and bladder cancer: a case-control study. *J. Cell. Biochem.* 120 14847–14859. 10.1002/jcb.28746 31009110

[B75] Muñiz S. Gonzalvo P. Valdehita A. Molina-Molina J. M. Navas J. M. Olea N. (2017). Ecotoxicological assessment of soils polluted with chemical waste from lindane production: use of bacterial communities and earthworms as bioremediation tools. *Ecotoxicol. Environ. Saf.* 145 539–548. 10.1016/j.ecoenv.2017.07.070 28787615

[B76] Murthy H. M. R. Manonmani H. K. (2007). Aerobic degradation of technical hexachlorocyclohexane by a defined microbial consortium. *J. Hazard. Mater.* 149 18–25. 10.1016/j.jhazmat.2007.03.053 17502125

[B77] Nagata Y. Endo R. Ito M. Ohtsubo Y. Tsuda M. (2007). Aerobic degradation of lindane (γ-hexachlorocyclohexane) in bacteria and its biochemical and molecular basis. *Appl. Microbiol. Biotechnol.* 76 741–752. 10.1007/s00253-007-1066-x 17634937

[B78] Nagpal V. Paknikar K. (2006). Integrated biological approach for the enhanced degradation of lindane. *J. Basic Microbiol.* 56 820–826. 10.1002/jobm.201500559 26648050

[B79] Nagpal V. Srinivasan M. Paknikar K. (2008). Biodegradation of γ-hexachlorocyclohexane (Lindane) by a non-white rot fungus Conidiobolus 03-1-56 isolated from litter. *Indian J. Microbiol.* 48 134–141. 10.1007/s12088-008-0013-6 23100707 PMC3450201

[B80] Nanasato Y. Namiki S. Oshima M. Moriuchi R. Konagaya K.-I. Seike N. (2016). Biodegradation of γ-hexachlorocyclohexane by transgenic hairy root cultures of *Cucurbita moschata* that accumulate recombinant bacterial LinA. *Plant Cell Rep.* 35 1963–1974. 10.1007/s00299-016-2011-1 27295266

[B81] Nolan K. Kamrath J. Levitt J. (2012). Lindane toxicity: a comprehensive review of the medical literature. *Pediatr. Dermatol.* 29 141–146. 10.1111/j.1525-1470.2011.01519.x 21995612

[B82] Padhi S. Pati B. (2016). Severity of persistence and toxicity of hexachlorocyclohexane (HCH) to the environment-A current approach. *Scholarly Res. J. Interdiscip. Stud.* 4 3158–3168.

[B83] Pamela C. Andrea S. D. Nadin U. Uwe S. T. Albrecht P. Gerrit S. (2011). Determination of lindane leachability in soil-biosolid systems and its bioavailability in wheat plants. *Chemosphere* 84 397–402. 10.1016/j.chemosphere.2011.03.070 21524779

[B84] Polti M. A. Aparicio J. D. Benimeli C. S. Amoroso M. J. (2014). Simultaneous bioremediation of Cr (VI) and lindane in soil by actinobacteria. *Int. Biodeterior. Biodegrad.* 88 48–55. 10.1016/j.ibiod.2013.12.004

[B85] Quintero J. C. Lu-Chau T. A. Moreira M. T. Feijoo G. Lema J. M. (2007). Bioremediation of HCH present in soil by the white-rot fungus *Bjerkandera adusta* in a slurry batch bioreactor. *Int. Biodeterior. Biodegrad.* 60 319–326. 10.1016/j.ibiod.2007.05.005

[B86] Quintero J. C. Moreira M. T. Feijoo G. Lema J. M. (2005a). Anaerobic degradation of hexachlorocyclohexane isomers in liquid and soil slurry systems. *Chemosphere* 61 528–536. 10.1016/j.chemosphere.2005.02.010 16202806

[B87] Quintero J. C. Moreira M. T. Feijoo G. Lema J. M. (2005b). Effect of surfactants on the soil desorption of hexachlorocyclohexane (HCH) isomers and their anaerobic biodegradation. *J. Chem. Technol. Biotechnol.* 80 1005–1015. 10.1002/jctb.1277

[B88] Rinku P. Shashi B. Mandeep D. Mukesh K. Gauri D. Om P. (2005). Hexachlorocyclohexane-degrading bacterial strains *Sphingomonas paucimobilis* B90A, UT26 and Sp+, having similar lin genes, represent three distinct species, *Sphingobium indicum* sp. nov., *Sphingobium japonicum* sp. nov. and *Sphingobium francense* sp. nov., and reclassification of [Sphingomonas] chungbukensis as *Sphingobium chungbukense* comb. nov. *Int. J. Syst. Evol. Microbiol.* 55 1965–1972. 10.1099/ijs.0.63201-0 16166696

[B89] Saez J. Alvarez A. Fuentes M. Amoroso M. Benimeli C. (2017). “An overview on microbial degradation of lindane,” in *Microbe-Induced Degradation of Pesticides*, ed. Singh S., (Cham: Springer), 191–212. 10.1007/978-3-319-45156-5_9

[B90] Saez J. M. Aparicio J. D. Amoroso M. J. Benimeli C. S. (2015). Effect of the acclimation of a *Streptomyces* consortium on lindane biodegradation by free and immobilized cells. *Process Biochem.* 50 1923–1933. 10.1016/j.procbio.2015.08.014

[B91] Saez J. M. Bigliardo A. L. Raimondo E. E. Briceño G. E. Polti M. A. Benimeli C. S. (2018). Lindane dissipation in a biomixture: effect of soil properties and bioaugmentation. *Ecotoxicol. Environ. Saf.* 156 97–105. 10.1016/j.ecoenv.2018.03.011 29533212

[B92] Sagar V. Singh D. (2011). Biodegradation of lindane pesticide by non white-rots soil fungus *Fusarium* sp. *World J Microbiol. Biotechnol.* 27 1747–1754. 10.1007/s11274-010-0628-8

[B93] Sahoo B. Ningthoujam R. Chaudhuri S. (2019). Isolation and characterization of a lindane degrading bacteria *Paracoccus* sp. NITDBR1 and evaluation of its plant growth promoting traits. *Int. Microbiol.* 22 155–167. 10.1007/s10123-018-00037-1 30810939

[B94] Sainz M. J. Gonzalez-Penalta B. Vilarino A. (2010). Effects of hexachlorocyclohexane on rhizosphere fungal propagules and root colonization by arbuscular mycorrhizal fungi in *Plantago lanceolata*. *Eur. J. Soil Sci.* 57 83–90. 10.1111/j.1365-2389.2005.00775.x

[B95] Salam J. A. Das N. (2015). Degradation of lindane by a novel embedded bio-nano hybrid system in aqueous environment. *Appl. Microbiol. Biotechnol.* 99 2351–2360. 10.1007/s00253-014-6112-x 25304880

[B96] Salam J. A. Hatha M. A. Das N. (2017). Microbial-enhanced lindane removal by sugarcane (*Saccharum officinarum*) in doped soil-applications in phytoremediation and bioaugmentation. *J. Environ. Manage.* 193 394–399. 10.1016/j.jenvman.2017.02.006 28259469

[B97] Salam J. A. Lakshmi V. Das D. Das N. (2013). Biodegradation of lindane using a novel yeast strain, *Rhodotorula* sp. VITJzN03 isolated from agricultural soil. *World J. Microbiol. Biotechnol.* 29 475–487. 10.1007/s11274-012-1201-4 23108665

[B98] Sangwan N. Lata P. Dwivedi V. Singh A. Niharika N. Kaur J. (2012). Comparative metagenomic analysis of soil microbial communities across three hexachlorocyclohexane contamination levels. *PLoS One* 7:e46219. 10.1371/journal.pone.0046219 23029440 PMC3460827

[B99] Sharma P. Shankar S. Agarwal A. Singh R. (2010). Variation in serum lipids and liver function markers in lindane exposed female wistar rats: attenuating effect of curcumin, vitamin C and vitamin E. *Asian J. Exp. Biol. Sci.* 1 440–444.

[B100] Shong J. Diaz M. R. J. Collins C. H. (2012). Towards synthetic microbial consortia for bioprocessing. *Curr. Opin. Biotechnol.* 23 798–802. 10.1016/j.copbio.2012.02.001 22387100

[B101] Sineli P. E. Tortella G. Costa J. D. Benimeli C. S. Cuozzo S. A. (2016). Evidence of α-, β-and γ-HCH mixture aerobic degradation by the native actinobacteria *Streptomyces* sp. M7. *World J. Microbiol. Biotechnol*. 32:81. 10.1007/s11274-016-2037-0 27038951

[B102] Singh B. K. Kuhad R. C. (2000). Degradation of insecticide lindane (γ-HCH) by white-rot fungi *Cyathus bulleri* and Phanerochaete sordida. *Pest Manag. Sci.* 56 142–146. 10.1002/1526-4998(200002)56:2<142::aidps104>3.0.co;2-i

[B103] Singh R. Manickam N. Mudiam M. K. R. Murthy R. C. Misra V. (2013). An integrated (nano-bio) technique for degradation of γ-HCH contaminated soil. *J. Hazard. Mater.* 258 35–41. 10.1016/j.jhazmat.2013.04.016 23692681

[B104] Singh T. Singh D. K. (2019a). Lindane degradation by root epiphytic bacterium *Achromobacter* sp. strain A3 from *Acorus calamus* and characterization of associated proteins. *Int. J. Phytoremediat.* 21 419–424. 10.1080/15226514.2018.1524835 30648424

[B105] Singh T. Singh D. K. (2019b). Rhizospheric *Microbacterium* sp. P27 showing potential of lindane degradation and plant growth promoting traits. *Curr. Microbiol.* 76 888–895. 10.1007/s00284-019-01703-x 31093691

[B106] Sun G. Du Y. Yin J. Jiang Y. Zhang D. Jiang B. (2019). Response of microbial communities to different organochlorine pesticides (O) contamination levels in contaminated soils. *Chemosphere* 215 461–469. 10.1016/j.chemosphere.2018.09.160 30336323

[B107] Tsygankov V. Y. Lukyanova O. Boyarova M. Gumovskiy A. Donets M. Lyakh V. (2019). Organochlorine pesticides in commercial Pacific salmon in the Russian Far Eastern seas: food safety and human health risk assessment. *Mar. Pollut. Bull.* 140 503–508. 10.1016/j.marpolbul.2019.02.008 30803671

[B108] Usman M. Tascone O. Faure P. Hanna K. (2014). Chemical oxidation of hexachlorocyclohexanes (HCHs) in contaminated soils. *Sci. Total Environ.* 476 434–439. 10.1016/j.scitotenv.2014.01.027 24486498

[B109] Vankar P. S. Ramashanker S. (2011). Lindane levels in the dumping grounds in Chinhat area, Lucknow, India. *Elecron. J. Environ. Agric. Food. Chem.* 10 2081–2089.

[B110] Vega M. Romano D. Uotila E. (2016). *Lindane (persistent organic pollutant) in the EU. Directorate General for Internal Policies. Policy Department C: Citizens’ Rights and Constitutional Affairs. Petitions. PE 571.398*. Available at: https://www.europarl.europa.eu/RegData/etudes/STUD/2016/571398/IPOL_STU(2016)571398_EN.pdf (accessed August 16, 2019).

[B111] Vijgen J. Abhilash P. Li Y. F. Lal R. Forter M. Torres J. (2011). Hexachlorocyclohexane (HCH) as new Stockholm Convention POPs—a global perspective on the management of Lindane and its waste isomers. *Environ. Sci. Pollut. Res.* 18 152–162. 10.1007/s11356-010-0417-9 21104204

[B112] Vijgen J. de Borst B. Weber R. Stobiecki T. Forter M. (2019). HCH and lindane contaminated sites: European and global need for a permanent solution for a long-time neglected issue. *Environ. Pollut.* 248 696–705. 10.1016/j.envpol.2019.02.029 30849587

[B113] Vijgen J. Weber R. Lichtensteiger W. Schlumpf M. (2018). The legacy of pesticides and POPs stockpiles—a threat to health and the environment. *Environ. Sci. Pollut. Res.* 25 31793–31798. 10.1007/s11356-018-3188-3 30280348

[B114] Wacławek S. Antoš V. Hrabák P. Černík M. (2016). Remediation of hexachlorocyclohexanes by cobalt-mediated activation of peroxymonosulfate. *Desalin. Water Treat.* 57 26274–26279. 10.1080/19443994.2015.1119757

[B115] Wang C. W. Chang S. C. Liang C. (2019). Persistent organic pollutant lindane degradation by alkaline cold-brew green tea. *Chemosphere* 232 281–286. 10.1016/j.chemosphere.2019.05.187 31154189

[B116] Wang S. M. Tseng S. K. (2009). Reductive dechlorination of trichloroethylene by combining autotrophic hydrogen-bacteria and zero-valent iron particles. *Bioresour. Technol.* 100 111–117. 10.1016/j.biortech.2008.05.033 18603424

[B117] Wang X. L. (2019). Joint multi-departmental submission: prohibition on production, circulation, application, import and export of pesticides such as lindane. *Mod. Agrochem.* 18:44.

[B118] Weber R. Aliyeva G. Vijgen J. (2013). The need for an integrated approach to the global challenge of POPs management. *Environ. Sci. Pollut. Res.* 20 1901–1906. 10.1007/s11356-012-1247-8 23143820

[B119] Yang J. Feng Y. Zhan H. Liu J. Yang F. Zhang K. (2018). Characterization of a pyrethroid-degrading *Pseudomonas fulva* strain P31 and biochemical degradation pathway of D-phenothrin. *Front. Microbiol.* 9:1003. 10.3389/fmicb.2018.01003 29867894 PMC5964208

[B120] Ye T. Zhou T. Li Q. Xu X. Fan X. Zhang L. (2020). *Cupriavidus* sp. HN-2, a novel quorum quenching bacterial isolate, is a potent biocontrol agent against *Xanthomonas campestris* pv. *campestris*. *Microorganisms* 8:E45. 10.3390/microorganisms8010045 31881662 PMC7022395

[B121] Ye T. Zhou T. Fan X. Bhatt P. Zhang L. Chen S. (2019). *Acinetobacter lactucae* strain QL-1, a novel quorum quenching candidate against bacterial pathogen *Xanthomonas campestris* pv. *campestris*. *Front. Microbiol.* 10:2867 10.3389/fmicb.2019.02867 PMC6929412 31921047

[B122] Zhang H. Chen F. Zhao H. Lu J. Zhao M. Hong Q. (2018). Colonization on cucumber root and enhancement of chlorimuron-ethyl degradation in the rhizosphere by *Hansschlegelia zhihuaiae* S113 and root exudates. *J. Agric. Food Chem.* 66 4584–4591. 10.1021/acs.jafc.8b00041 29672047

[B123] Zhan H. Huang Y. Lin Z. Bhatt P. Chen S. (2020). New insights into the microbial degradation and catalytic mechanism of synthetic pyrethroids. *Environ. Res.* 182:109138. 10.1016/j.envres.2020.109138 32069744

[B124] Zhan H. Feng Y. Fan X. Chen S. (2018a). Recent advances in glyphosate biodegradation. *Appl. Microbiol. Biotechnol.* 102 5033–5043. 10.1007/s00253-018-9035-0 29705962

[B125] Zhan H. Wang H. Liao L. Feng Y. Fan X. Zhang L. (2018b). Kinetics and novel degradation pathway of permethrin in *Acinetobacter baumannii* ZH-14. *Front. Microbiol.* 9:98. 10.3389/fmicb.2018.00098 29456525 PMC5801723

[B126] Zhang J. Lu L. Chen F. Chen L. Yin J. Huang X. (2018). Detoxification of diphenyl ether herbicide lactofen by *Bacillus* sp. Za and enantioselective characteristics of an esterase gene lacE. *J. Hazard. Mater.* 341 336–345. 10.1016/j.jhazmat.2017.07.064 28802244

[B127] Zhang W. Ye Y. Hu D. Ou L. Wang X. (2010). Characteristics and transport of organochlorine pesticides in urban environment: air, dust, rain, canopy throughfall, and runoff. *J. Environ. Monit.* 12 2153–2160. 10.1039/c0em00110d 20931122

